# Application of artificial intelligence for automatic cataract staging based on anterior segment images: comparing automatic segmentation approaches to manual segmentation

**DOI:** 10.3389/fnins.2023.1182388

**Published:** 2023-04-20

**Authors:** Fan Gan, Hui Liu, Wei-Guo Qin, Shui-Lian Zhou

**Affiliations:** ^1^Medical College of Nanchang University, Nanchang, China; ^2^Department of Ophthalmology, Jiangxi Provincial People’s Hospital, The First Affiliated Hospital of Nanchang Medical College, Nanchang, China; ^3^Department of Cardiothoracic Surgery, The 908th Hospital of Chinese People’s Liberation Army Joint Logistic Support Force, Nanchang, China

**Keywords:** anterior segment images, artificial intelligence, cortical cataract, multi-feature fusion, automatic segmentation

## Abstract

**Purpose:**

Cataract is one of the leading causes of blindness worldwide, accounting for >50% of cases of blindness in low- and middle-income countries. In this study, two artificial intelligence (AI) diagnosis platforms are proposed for cortical cataract staging to achieve a precise diagnosis.

**Methods:**

A total of 647 high quality anterior segment images, which included the four stages of cataracts, were collected into the dataset. They were divided randomly into a training set and a test set using a stratified random-allocation technique at a ratio of 8:2. Then, after automatic or manual segmentation of the lens area of the cataract, the deep transform-learning (DTL) features extraction, PCA dimensionality reduction, multi-features fusion, fusion features selection, and classification models establishment, the automatic and manual segmentation DTL platforms were developed. Finally, the accuracy, confusion matrix, and area under the receiver operating characteristic (ROC) curve (AUC) were used to evaluate the performance of the two platforms.

**Results:**

In the automatic segmentation DTL platform, the accuracy of the model in the training and test sets was 94.59 and 84.50%, respectively. In the manual segmentation DTL platform, the accuracy of the model in the training and test sets was 97.48 and 90.00%, respectively. In the test set, the micro and macro average AUCs of the two platforms reached >95% and the AUC for each classification was >90%. The results of a confusion matrix showed that all stages, except for mature, had a high recognition rate.

**Conclusion:**

Two AI diagnosis platforms were proposed for cortical cataract staging. The resulting automatic segmentation platform can stage cataracts more quickly, whereas the resulting manual segmentation platform can stage cataracts more accurately.

## 1. Introduction

Cataract is one of the leading causes of blindness worldwide, accounting for over 50% of cases of blindness in low- and middle-income countries (Wu et al., 2019). It is a visual impairment characterized by cloudiness or opacification of the crystalline lens, and most cataracts are age-related, although they can also be attributed to disease, trauma, or congenital factors (Do et al., 2013; Gao et al., 2015; Satyam et al., 2015). The pathogenesis of cataract is quite complex and results from the long-term comprehensive effect of various internal and external factors on the lens. Surgical removal of the lens and implantation of intraocular lens are the only effective treatments of a visually significant cataract (Son et al., 2022).

Cortical cataract is the most common type of the senile (age-related) cataract. Depending on its severity, cortical cataract is divided into four stages: (1) incipient stage, in which the lens is partially opaque, with spokes and vacuoles, and wedge-shaped opacity; (2) intumescent stage (immature stage), during which lens thickness is increased and the depth of the anterior chamber becomes shallow; (3) mature stage, in which the lens is completely opaque; and (4) the hypermature stage, which has a shrunken and wrinkled anterior capsule owing to water leakage out of the lens and might also have calcium deposits. In the incipient stage, because the lesion rarely involves the pupil area, vision is affected rarely. Some measures can be taken to slow cataract progression, such as by wearing anti-glare sunglasses (Gao et al., 2015). In the intumescent stage, for patients with anatomic factors of angle-closure glaucoma, an acute glaucoma attack can be induced by anterior chamber shallowing. By the mature stage, the patient will have severe vision loss and will require surgical treatment. In the hypermature stage, patients will have serious complications, such as phacolytic glaucoma and phacoanaphylactic uveitis. Therefore, for timely cataract treatment, to prevent complications, and to improve quality of life, accurate staging is important.

Currently, the diagnosis of cataract relies on the rich experience of the ophthalmologist and slit-lamp biomicroscopy examination. However, the distribution of medical resources is far from satisfactory for cataract diagnosis and management (Wu et al., 2019). The COVID-19 pandemic has also led to a shift from on-site medical needs to telemedicine. In the previous research, Xie et al. (2020) applied a semiautomated telemedicine platform combining a deep learning system with human assessment to achieve the best economic return for diabetic retinopathy (DR) screening in Singapore, resulting in potential savings of approximately 20% of the annual cost. Therefore, it is particularly important to develop an artificial intelligence (AI) diagnosis platform for cataracts to achieve high-precision automated diagnosis and lay the foundation for the combination of AI and telemedicine in the future.

Recently, artificial intelligence (AI) has made remarkable progress in medicine (Amjad et al., 2021). An increasing number of AI diagnostic models for ophthalmologic diseases have been proposed. Lin et al. (2020) used the random forest (RF) and adaptive boosting (Ada) algorithms for the identification of congenital cataracts. Gao et al. (2015) used deep-learning algorithms to grade nuclear cataracts. Hasan et al. (2021) used a transfer-learning algorithm to detect cataracts. All these models exhibit excellent performance. However, to the best of our knowledge, there has not been research applying AI for automatic cortical cataract staging. Most previous studies used the traditional machine learning or deep learning based on original slit-lamp images. Compared with traditional methods, transfer-learning represents an important way of solving the fundamental problem of insufficient training data in deep learning (He et al., 2020). In addition, for similar experimental conditions, a pre-trained network can be adjusted quickly through transfer-learning, which can reduce the training time greatly (Lin et al., 2021). It has also been suggested that the image features derived from segmented images yield increased accuracy than those from non-segmented images (Zhang et al., 2020). Automatic segmentation can be faster and more reproducible compared with manual delineation but might not have the same accuracy as manual segmentation (Huang et al., 2019; Tsuji et al., 2020).

**FIGURE 1:**
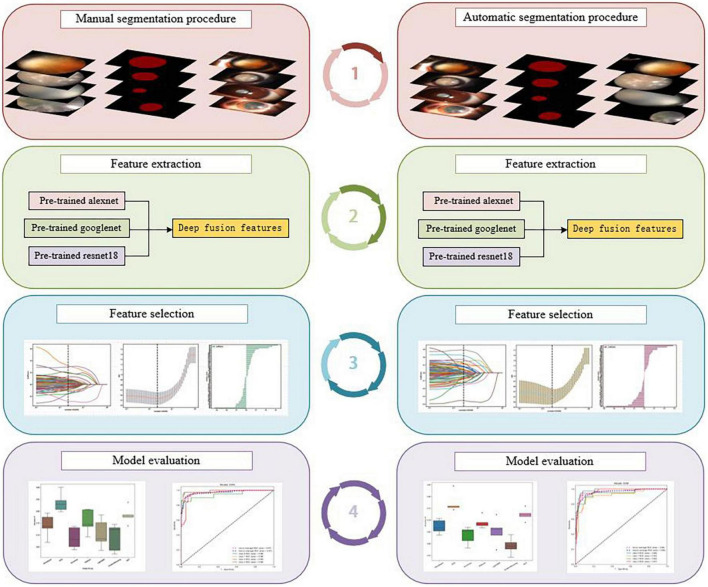
The flowchart of the detailed processes of the study: manual segmentation procedure **(left)**, automatic segmentation procedure **(right)**.

Therefore, unlike previous studies, we combined segmentation with a deep transfer-learning algorithm and multi-feature fusion to create two AI platforms for automatic cortical cataract staging. One is based on an automatic segmentation method, whereas the other is based on a manual segmentation method; the flowchart of the detailed processes within this study is shown in Figure 1.

## 2. Materials and methods

### 2.1. Data collection

We collected the anterior segment images of cataract-affected eyes from the Department of Ophthalmology, Jiangxi Provincial People’s Hospital. All images were diffuse-illuminated photographs that were collected from the same slit-lamp digital microscopy and taken by experienced ophthalmic technologists using standardized techniques. All images were screened, the images that clearly demonstrate the characteristics and reflect different stages of cataracts were retained, and blurry images and images of corneal disease that affected lens observation significantly were excluded. The screened high-quality images were then divided randomly into a training and test set using a stratified random-allocation technique at a ratio of 8:2, in which stratification was by staging of cortical cataracts. This means the distribution of data in each stage was random in both the training and testing sets.

### 2.2. The region of interest delineating and cataract labeling

An experienced ophthalmologist used the LabelMe software to delineate the region of interest (ROI), which was the lens regions of the images. Based on the diagnostic reports obtained from the electronic medical record system and combined with the opacity of the lens in the images, the ROIs were labeled as “label 0”, indicating the incipient stage, “label 1”, indicating the intumescent stage (immature stage), “label 2”, indicating the mature stage, and “label 3” indicating the hypermature stage.

### 2.3. Establishment of the automatic segmentation DTL platform

#### 2.3.1. Establishment of the automatic segmentation model

First, we trained the automatic segmentation model with the FCNResnet50 architecture. The FCN model ‘learns’ a pixel’s class by finding optimal values for the model parameters through minimizing the prediction error against the target data set (Larsen et al., 2021). The images that the experienced ophthalmologist had delineated the lens regions of were then used as the gold standard. The model was run for 30 ‘epochs’, each time training on 80% of the dataset and evaluating model performance on a 20% hold-out set. Finally, the trained model was applied to the whole dataset and the segmentations of lens regions were obtained.

#### 2.3.2. Deep fusion features extraction

First, the alexNet, googleNet, and resnet18 models were pretrained on the natural image dataset ImageNet dataset^1^, respectively. Then the pretraining parameters obtained from the ImageNet dataset were used to initialize our models. The resulting pretrained alexNet, googleNet, and resnet18 models were then utilized to extract DTL features from the output of the avgPool layer, respectively. To reduce dimensionality, we employed principal components analysis (PCA). Subsequently, we utilized channel concat to combine the output features after PCA dimension reduction, and this resulted in the deep fusion features.

#### 2.3.3. Feature selection

The final deep fusion features used to construct the model were selected in the training set. The least absolute shrinkage and selection operator (LASSO) algorithm was used to construct the feature selection model. First, all the deep fusion features were standardized to a mean of 0 and a variance of 1 by the regularization method. The formula used is shown here:


$$c\,o\,l\,u\,m\,n=\frac{\text{column}-\text{mean}}{\text{std}}$$

Then, the LASSO model selected features using a tuning parameter (λ). The optimal λ was chosen based on a ten-fold cross-validation. Depending on the regulation weight λ, LASSO shrinks all regression coefficients toward zero and sets the coefficients of many irrelevant features exactly to zero (Lao et al., 2017). The features with non-zero coefficients were retained.

#### 2.3.4. Establishment of the classification model

After features selection, the selected features were used to establish the classification models. Seven machine-learning algorithms were imported from the scikit-learn python library to establish seven classification models, respectively, including naive bayes (NB), support vector machines (SVM), extremely randomized trees (Extra Trees, ET), extreme gradient boosting (XGBoost, XGB), light gradient boosting machine (LightGBM), gradient boosting (GB), and multilayer perceptron (MLP) models. To prevent overfitting, five-fold cross-validation was used to fit each classification model.

### 2.4. Establishment of the manual segmentation DTL platform

Manual segmentation of the DTL platform included manual segmentation, deep fusion features extraction, feature selection, and the classification model establishment.

The rest was the same as the automatic segmentation DTL platform, except that the segmentation was different. First, based on the lens regions delineated by an experienced ophthalmologist, a ROI was segmented manually from each image. Then, based on the ROI, as with automatic segmentation DTL platform, pretrained alexNet, pretrained googleNet, and pretrained resNet18 models were used to extract DTL features, respectively. Next, PCA was used for dimensionality reduction. The reduced DTL features were fused. The LASSO model was used to select features. Finally, seven different classification models, NB, SVM, ET, XGB, LightGBM, GB, and MLP were established.

### 2.5. Model validation and performance evaluation

The trained models were applied to the test set for independent testing. Different quantitative metrics, such as pixel accuracy (PA), intersection over union (IoU), and Dice coefficient (Dice), were adopted to evaluate the performance of the automatic segmentation model and the classification model. PA is the simplest indicator of image segmentation, which is the percentage of correctly classified pixels out of the total pixels in each image (Larsen et al., 2021). IoU is a concept used in object detection, which measures the overlap between two boundaries: the predicted boundary and the truth boundary (Kim and Hidaka, 2021). The higher the IoU, the more accurate is the position of the prediction boundary. The Dice coefficient is a score that indicates the similarity between two samples (Takahashi et al., 2021). It used to measure the amount of overlap of regions.

To the classification models, the classification accuracy, confusion matrix, and the receiver operating characteristic (ROC) curve and area under the ROC curve (AUC) were also introduced to evaluate the performance. The classification accuracy is computed as the ratio of the correctly classified number of samples and the total number of samples (Masood and Farooq, 2019). The confusion matrix is a visualization tool used typically in multiclass supervised learning and contains information about the actual classifications and the classifications predicted by a classification model (Bang et al., 2021). ROC curve and AUC was another class of indicators to evaluate the classification accuracy. The closer the ROC curve is to the upper left corner, the larger the AUC value, and the better the classification effect.

### 2.6. Statistical analysis

ROI was delineated, segmented, and labeled using an open-source annotation tool LabelMe. All statistical calculations and the drawing of statistical graphs were performed in Python (version 3.9.7).

## 3. Results

### 3.1. Imaging dataset

A total of 647 high quality anterior segment images were included into the dataset. One hundred ninety one incipient stage images, 171 intumescent stage images, 100 mature stage images and 183 hypermature stage images. Through stratified random division, with 80% of the images used for training and 20% for testing. 517 images were included in training set, of these, included 153 incipient stage images, 136 intumescent stage images, 80 mature stage images and 147 hypermature stage images. One hundred thirty images were included in test set, of these, included 38 incipient stage images, 35 intumescent stage images, 20 mature stage images and 36 hypermature stage images.

### 3.2. Segmentation performance of the automatic segmentation model

**FIGURE 2:**
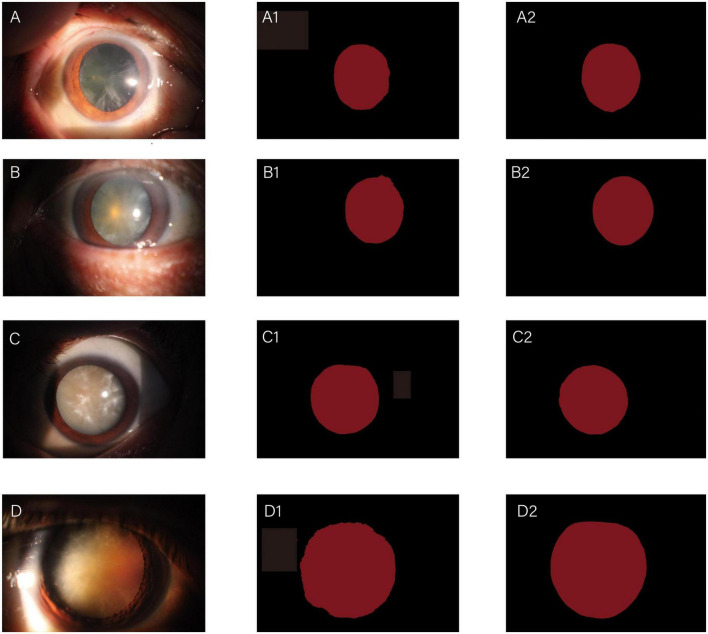
Diagrams of comparisons between automatic and manual segmentation: original images of cataracts at different stage **(A–D)**; the corresponding automatic segmentation mask **(A1–D1)**; the corresponding manual segmentation mask **(A2–D2)**.

**FIGURE 3:**
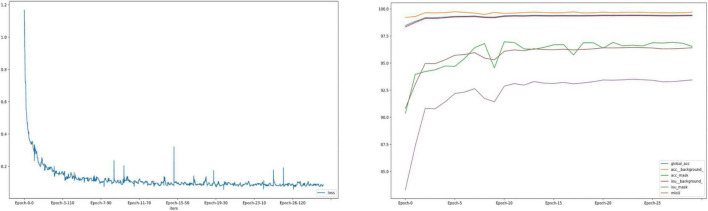
The loss convergence and accuracy curves of FCNResNet50 model in the test. The loss convergence curve **(left)**, the pixel accuracy (PA) and Intersection over Union (IoU) curve **(right)**.

The whole automatic segmentation process took 2 min and 43 s. While the manual segmentation process from the experienced ophthalmologist required approximately a week. The segmentation results graph of the automated and manual segmentations as shown in Figure 2. The visualization of the FCNResnet50 model training process was shown in Figure 3. The loss value decreases gradually with epoch and stabilizes at 5 epochs, and the accuracy reaches 95% in the test set. It can be seen that the contours obtained manually often fit better with the true contour of the lens compared with the automatic segmentation. And the results of automatic segmentation showed that the PA was 98.9, the mean IoU was 93.3, and the mean Dice score was 96.4%.

### 3.3. Results of the feature extraction and fusion

In the automatic segmentation platform, the automatic segmentation lens images were input to the three pretrained models, the extracted features were output from the last fully connected layer. 512, 1024, 9216 DTL features of each image were extracted from pre-trained alexNet, pre-trained googleNet and pre-trained resNet 18, respectively. After PCA dimension reduction, 31 features of each image from each model were obtained. And then after features fusion, the feature subset included 93 features of each image were obtained. In the manual segmentation platform, the manual segmentation lens images were input to the three pretrained models. After features extraction, PCA dimension reduction and features fusion, the 93 features of each image were also obtained.

### 3.4. Results of feature selection

**FIGURE 4:**
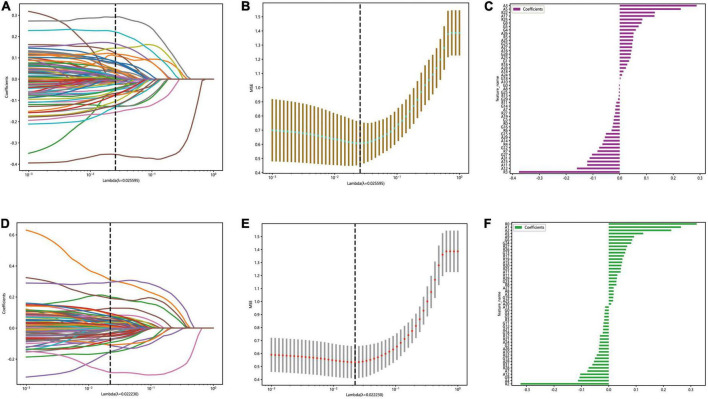
The figure of LASSO coefficient distribution (left): the colored curve shows the path of the coefficients for each input feature as lambda varies; the figure of partial likelihood deviation of the LASSO coefficient distribution (middle): the vertical dashed line represents the optimal value of the regularization parameter determined by cross-validation; feature weight coefficient graph (right). Automatic segmentation platform **(A–C)**; manual segmentaion platform **(D–F)**.

The optimal λ (λ = 0.025595) was chosen based on a ten-fold cross-validation. Depending on the optimal λ, 49 features were retained in the automatic segmentation platform, including 21 features of alexNet model, 12 features of resNet model and 16 features of googleNet model. 51 features were retained in the manual segmentation platform, including 20 features of alexnet model, 17 features of resNet model and 14 features of googleNet model. The selection process was shown in Figure 4.

### 3.5. The classification performance of the automatic segmentation DTL platform and the manual segmentation DTL platform

**FIGURE 5:**
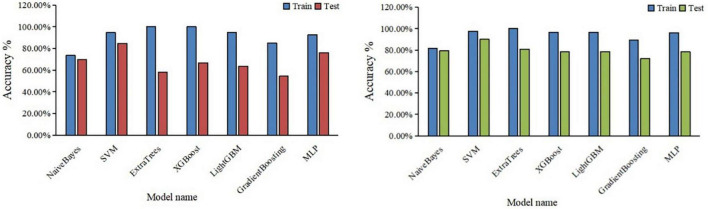
Boxplots for the range of accuracy of each classification model, the automatic segmentation platform **(left)**; the manual segmentation platform **(right)**.

By five-fold cross-validation, the result of classification accuracy revealed that the accuracy of SVM model was the best in both the automatic segmentation DTL platform or the manual segmentation DTL platform. In the automatic segmentation DTL platform, the accuracy of the model in the training set and the test set were 94.59 or 84.50%, respectively. In the manual segmentation DTL platform, the accuracy of the model in the training set and the test set were 97.4 or 90.00%, as shown in Table 1. The range of classification accuracy rates were shown in Figure 5.

**TABLE 1:** The accuracy of classification models in the automatic and manual segmentation DTL platforms.

Group	Model name	Accuracy	Train/Test
Manual	NaiveBayes	81.82%	Train
	NaiveBayes	79.23%	Test
	SVM	97.48%	Train
	SVM	90.00%	Test
	ExtraTrees	100%	Train
	ExtraTrees	80.77%	Test
	XGBoost	96.71%	Train
	XGBoost	78.46%	Test
	LightGBM	96.71%	Train
	LightGBM	78.46%	Test
	GradientBoosting	89.17%	Train
	GradientBoosting	72.31%	Test
	MLP	95.94%	Train
	MLP	78.46%	Test
Automatic	NaiveBayes	73.55%	Train
	NaiveBayes	69.77%	Test
	SVM	94.59%	Train
	SVM	84.50%	Test
	ExtraTrees	100%	Train
	ExtraTrees	58.14%	Test
	XGBoost	100%	Train
	XGBoost	66.67%	Test
	LightGBM	94.79%	Train
	LightGBM	63.57%	Test
	GradientBoosting	84.94%	Train
	GradientBoosting	54.26%	Test
	MLP	92.66%	Train
	MLP	75.97%	Test

**FIGURE 6:**
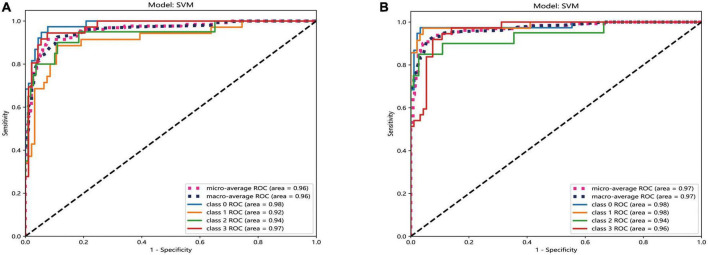
The ROC curves of SVM model of two different platforms in the test set. The automatic segmentation platform **(A)**; the manual segmentation platform **(B)**, “Class 0” indicated incipient stage, “class 1” indicated intumescent stage, “class 2” indicated mature stage, and “class 3” indicated hypermature stage.

The result of AUC also revealed that the performance of SVM model was best in both two platforms. In the automatic segmentation DTL platform, the micro and macro average AUC of SVM model both were 96% in the test set. In the manual segmentation DTL platform, the micro and macro average AUC of SVM model both were 97% in the test set. And in both two platforms, the AUC for each classification was all more that 90% in the test set. As shown in Figure 6.

**FIGURE 7:**
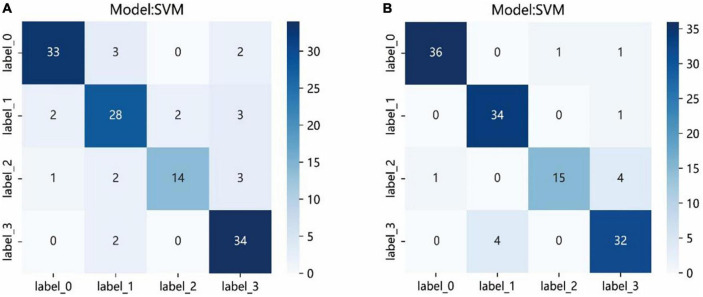
The confusion matrix of the SVM model of two different platforms in the test set. Each column represents the predicted class and each row of the matrix represents the actual class. The automatic segmentation platform **(A)**; the manual segmentation platform **(B)**. “Label 0” indicated incipient stage, “label 1” indicated intumescent stage, “label 2” indicated mature stage, and “label 3” indicated hypermature stage.

The results of 4 × 4 matrix show the number of correct and incorrect classifications by the SVM model in each stage of cataract. In the automatic segmentation DTL platform, the recognition rates of incipient stage, intumescent stage, hypermature stage were all high. The hypermature stage had the highest recognition rate. Of the 36 images, 34 of them were correctly recognized (94.5%) and only 2 were incorrectly recognized (5.5%). While, the mature stage had the lowest recognition rate. Of the 20 images, 14 of them were correctly recognized (70%) and 6 were incorrectly recognized (6%). In the manual segmentation DTL platform, the results also show that all stages except for mature had high recognition rate. Of the 20 images in the mature stage, 15 of them were correctly recognized (75%) and 5 were incorrectly recognized (5%). The recognition rate of the intumescent stage was highest, of the 35 images, 34 of them were correctly recognized (97.1%) and only 1 were incorrectly recognized (2.9%) as shown in Figure 7.

In addition to the SVM model, we also drew the ROC curves and confusion matrix of the other models, which included NB, ET, XGB, LightGBM, GB, and MLP models of the manual segmentation platform and the automatic segmentation platform. The ROC curves of other models were shown in the Supplementary Figures 1, 2. The confusion matrix of other models were shown in the Supplementary Figures 3, 4.

## 4. Discussion

Precision medicine is an emerging medical model that has great promise for the prevention, diagnosis, and treatment of many diseases (McGonigle, 2016). Accurate staging of cataracts is a precise classification of the different states and processes of a disease, which is the embodiment of precision medicine strategies. It is also essential to planning of appropriate treatment, assessing outcome, and future prognosis. The establishment of an automated cataract diagnosis platform not only makes medical services more convenient and efficient, but also contributes to epidemic prevention and control. In this study, we developed two AI platforms based on using a deep transfer-learning algorithm and a multi-feature fusion method. The results of our study indicated that both platforms can stage cataract well. In the automatic segmentation DTL platform, the segmentation process completed in just 2 min and 43 s, with training and test set accuracies of 94.59 and 84.50% respectively. On the other hand, the manual segmentation DTL platform required approximately a week for an experienced ophthalmologist to manually segment. However, the model achieved higher accuracies in the training and test sets, at 97.48 and 90.00%, respectively. On the whole, the manual segmentation DTL platform was more precise, whereas the automatic segmentation DTL platform was more rapid.

The grading of cataracts is based on the opacity of the lens, and good segmentation performance is the basis of classification. The difference in the tissue outside the lens might affect the classification results. In the automatic segmentation DTL platform, based on the FCNResnet50 model, we have presented a method for the automatic segmentation of the lens from cataract images. The segmentation results showed that the proposed model was able to segment the lens accurately. Compared with previous research, the PA, IoU, and Dice improved by 8.4, 14.9, and 9.5%, respectively (Cai et al., 2021).

In previous studies, Gao et al. (2015) used a deep learning method to grade nuclear cataracts, but the accuracy only reached 70%. Lin used a convolutional recursive neural network to develop an AI platform for diagnosing childhood cataracts and the accuracy was 87.4%, whereas the accuracy of our study reached 90.00%. In addition to the task itself, the reason is possibly caused by the algorithmic upgrading. In this study, we adopt three pretrained models trained on the ImageNet and then fine-tuned into our dataset, which makes up for the insufficient datasets and leads to a reduction in the learning time. We also adopt the early fusion approaches for the classification task. Early fusion is also called feature level fusion, which emphasizes data combination before the classification (Zhang et al., 2017), which reduced the influence of single feature inherent defects and realized feature complementarity. Multiple studies have also confirmed that the combination of different features presents better classification results than individual features (Fang et al., 2019; Wan and Tan, 2019; Nemoto et al., 2020).

Comparing the results of the ROC curves of the two platforms, the macro average calculates the indicators of each class independently and then takes the mean value to treat all classes equally; the micro average aggregates the contributions of all classes to calculate the average indicator (Huang et al., 2022). The results of the two platforms can reach >95%, indicating that both show good performance. The AUC for each classification was >90% in the test set, indicating that both platforms have excellent classification accuracy.

The confusion matrices showed the prediction results of each sample in the test set. Although the results showed that all stages, except for mature, had high recognition rate, the probabilities of correct identification (PCIs) of mature stage in the two platforms achieved >70%. The images of the mature stage were misassigned to the hypermature stage easily. The major reason for this result might be that the staging of the cataracts is determined by the opacity of the lens, also, sometimes it is hard to define clear boundaries of adjacent stage, and a large sample size might be required. Compared with other stages, the sample size of the mature stage was the smallest.

This study had some other limitations. All data are only based on the diffuse-illuminated photographs, it is important to note that slit-lamp photography, fundus photography, and clinical data can also provide valuable insights into the disease. And this study was only based on clinical diagnosis of the disease; however, individualized treatment is an integral and mandatory part of precision medicine. Therefore, in future studies, we will increase the sample size and combine multiple modal data to combine diagnosis and treatment, to build a more perfect and convenient AI platform for clinical diagnosis and treatment.

## 5. Conclusion

In this study, two AI diagnosis platforms have been proposed for cortical cataract staging. Through the multi-feature transfer-learning method combined with an automatic or manual segmentation algorithm, the resulting automatic segmentation platform can stage cataracts more quickly, whereas the resulting manual segmentation platform can stage cataracts more accurately.

## Data availability statement

The raw data supporting the conclusions of this article will be made available by the authors, without undue reservation.

## Ethics statement

The studies involving human participants were reviewed and approved by Declaration of Helsinki and was approved by the Medical Ethics Committee of the Jiangxi Provincial People’s Hospital. The patients/participants provided their written informed consent to participate in this study. Written informed consent was obtained from the individual(s) for the publication of any potentially identifiable images or data included in this article.

## Author contributions

FG, HL, W-GQ, and S-LZ contributed to data collection, statistical analyses, and wrote the manuscript. All authors read and approved the final manuscript, contributed to the manuscript and approved the submitted version.

## Footnotes

1
www.image-net.org/
