# Supplementary material for: Application of artificial intelligence for automatic cataract staging based on anterior segment images: comparing automatic segmentation approaches to manual segmentation
Source: Front Neurosci. 2023 Apr 20;17:1182388. doi: 10.3389/fnins.2023.1182388 (PMC10159175; doi:10.3389/fnins.2023.1182388)
Supplement: Supplementary file 1 [file Data_Sheet_1.docx]

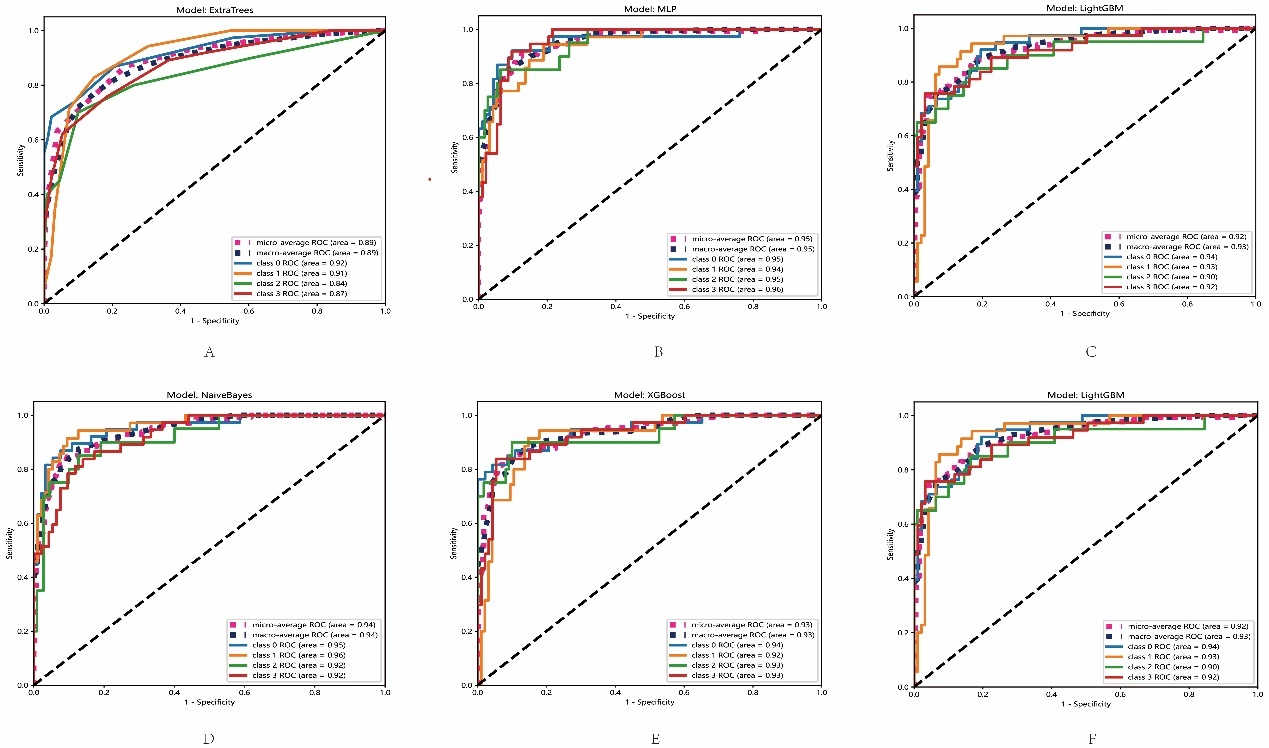


**Supplementary Figure 1**The ROC curves of different models of the manual segmentation platform in the test set. “class 0” indicated incipient stage, “class 1” indicated intumescent stage, “class 2” indicated mature stage, “class 3”indicated hypermature stage.


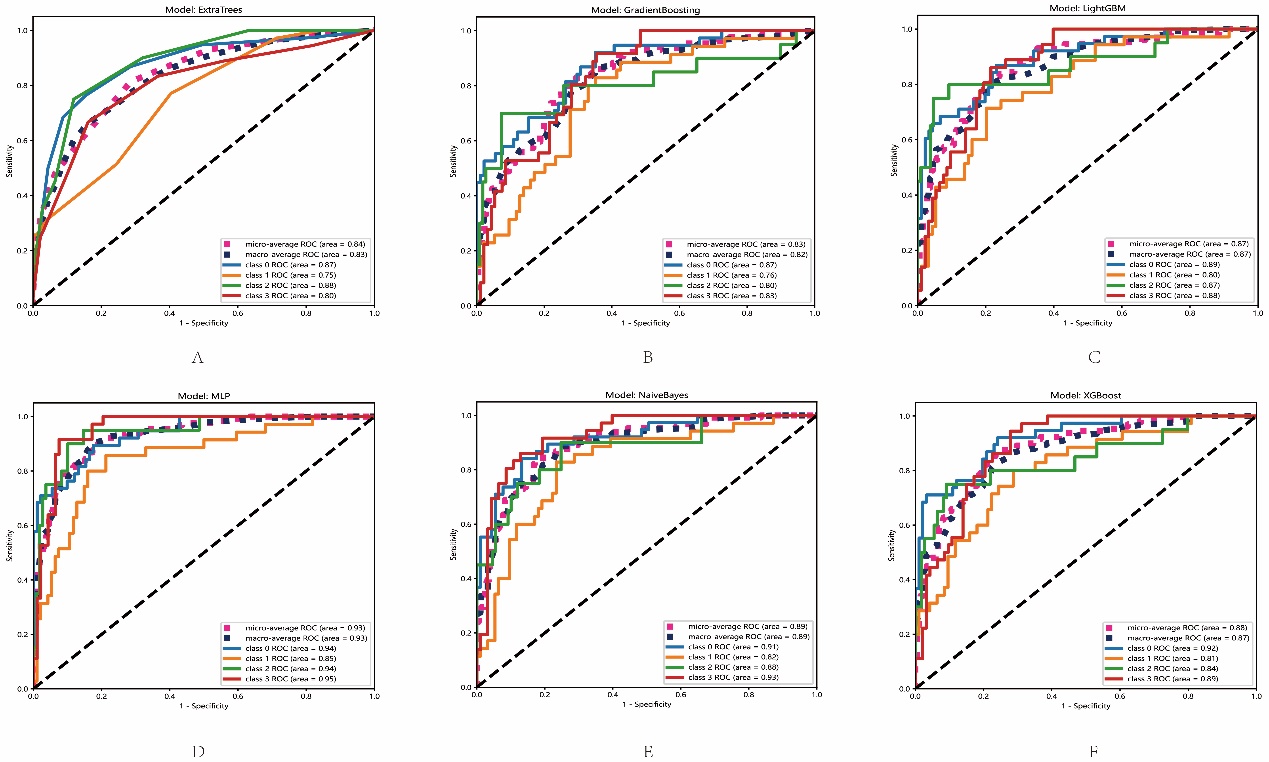


**Supplementary Figure 2** The ROC curves of different models of the automatic segmentation platform in the test set. “class 0” indicated incipient stage, “class 1” indicated intumescent stage, “class 2” indicated mature stage, “class 3” indicated hypermature stage.
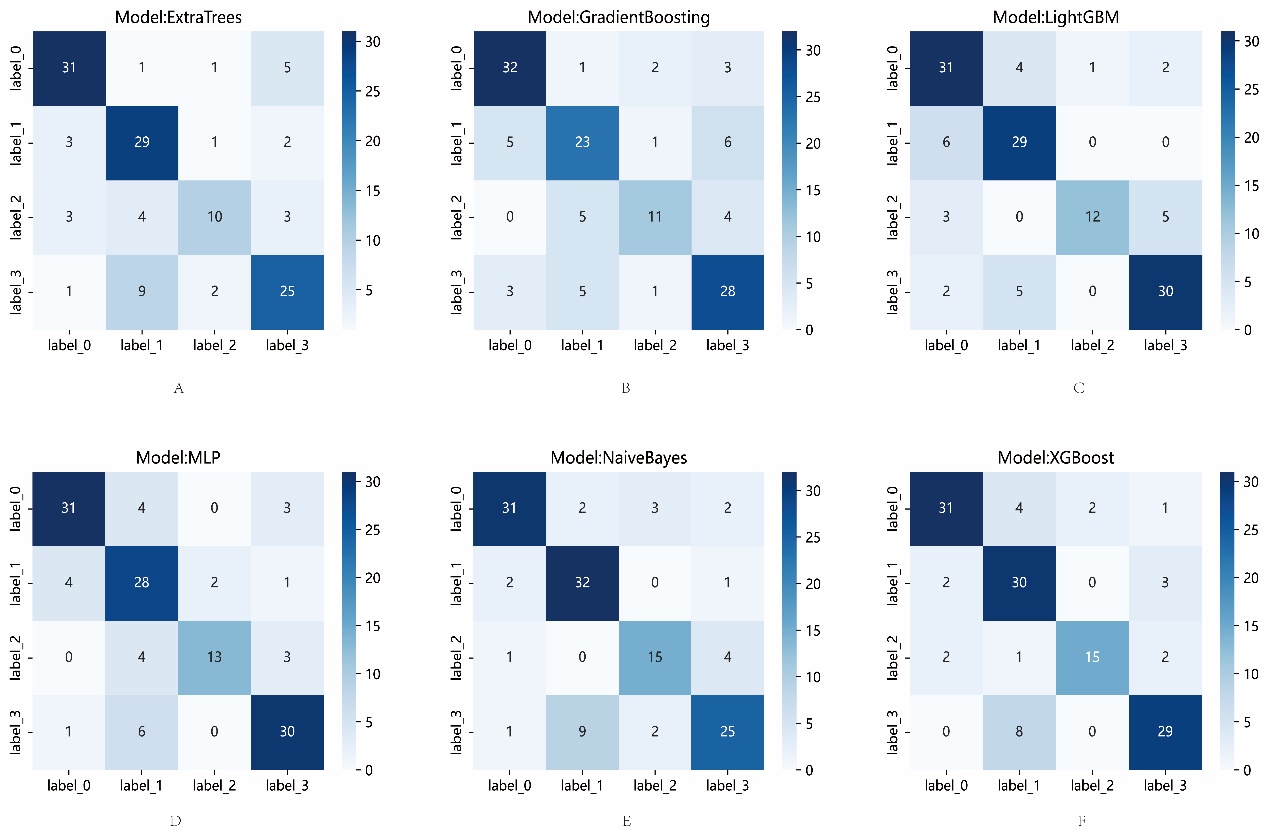


**Supplementary Figure 3** The confusion matrix of the different models of the manual segmentation platform in the test set. Each column represents the predicted class and each row of the matrix represents the actual class. “label 0” indicated incipient stage, “label 1” indicated intumescent stage, “label 2” indicated mature stage, “label 3” indicated hypermature stage.


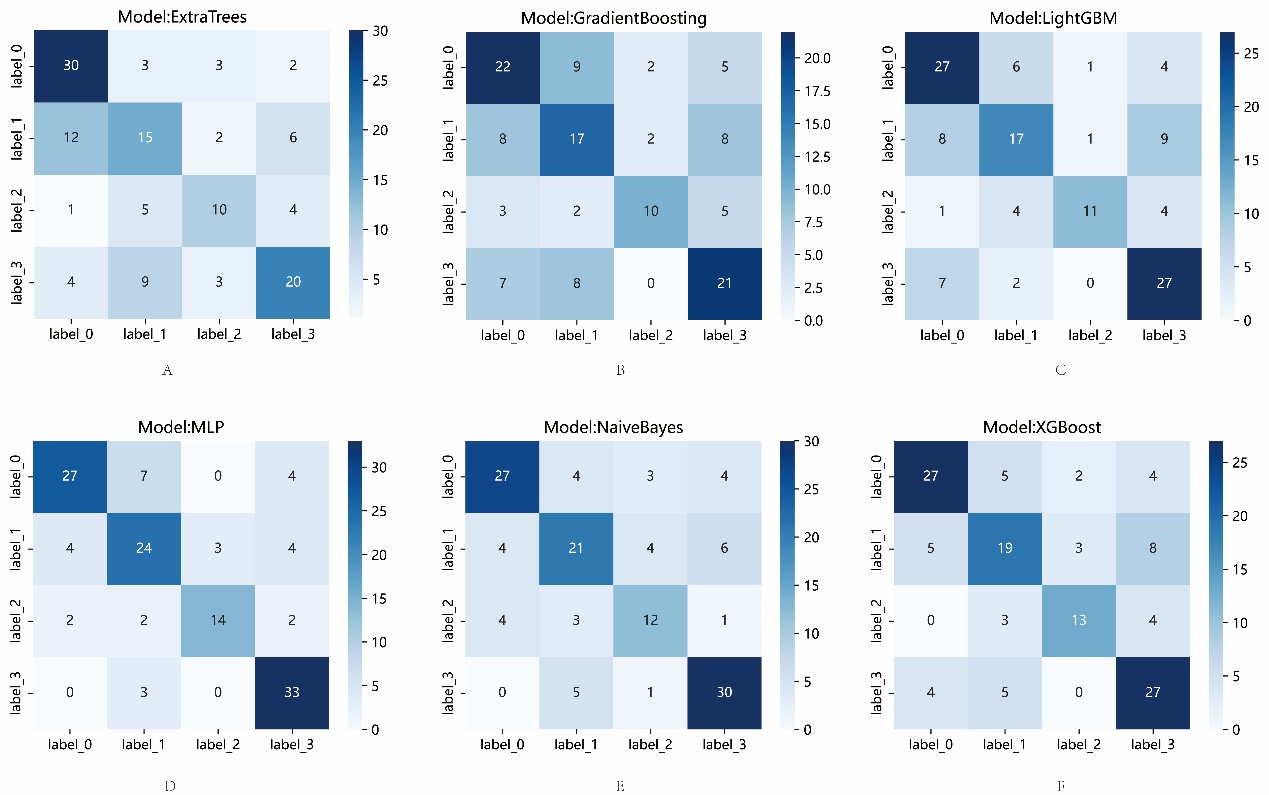


**Supplementary Figure 4** The confusion matrix of the different models of the automatic segmentation platform in the test set. Each column represents the predicted class and each row of the matrix represents the actual class. “label 0” indicated incipient stage, “label 1” indicated intumescent stage, “label 2” indicated mature stage, “label 3” indicated hypermature stage.
